# Brefeldin A Effectively Inhibits Cancer Stem Cell-Like Properties and MMP-9 Activity in Human Colorectal Cancer Colo 205 Cells

**DOI:** 10.3390/molecules180910242

**Published:** 2013-08-22

**Authors:** Chao-Neng Tseng, Chien-Fu Huang, Chung-Lung Cho, Hsueh-Wei Chang, Chao-Wei Huang, Chien-Chih Chiu, Yung-Fu Chang

**Affiliations:** 1 Graduate Institute of Natural Products, Department of Biomedical Science and Environmental Biology, Cancer Center, Kaohsiung Medical University Hospital, Kaohsiung Medical University, Kaohsiung 807, Taiwan; E-Mails: cntseng@kmu.edu.tw (C.-N.T.); changhw@kmu.edu.tw (H.-W.C.); 2 Department of Biological Sciences and Technology, I-Shou University, Kaohsiung 824, Taiwan; E-Mails: chienfu@isu.edu.tw (C.-F.H.); qqvictor@hotmail.com (C.-W.H.); 3 Department of Biological Sciences, National Sun Yat-Sen University, Kaohsiung 804, Taiwan; E-Mail: clcho@faculty.nsysu.edu.tw; 4 Department of Biotechnology, Kaohsiung Medical University, Kaohsiung 807, Taiwan; 5 Department of Biomedical Science and Environmental Biology, Kaohsiung Medical University, Kaohsiung 807, Taiwan

**Keywords:** brefeldin A, cancer stem cell, colorectal cancer, matrix metallopeptidase, clonogenesis

## Abstract

Cancer stem cells (CSCs) are a small subset of cancer cells with indefinite potential for self-renewal and the capacity to drive tumorigenesis. Brefeldin A (BFA) is an antibiotic that is known to block protein transport and induce endoplasmic reticulum (ER) stress in eukaryotic cells, but its effects on colorectal CSCs are unknown. We investigated the inhibitory effect of BFA on human colorectal cancer Colo 205 cells. We found that BFA effectively reduced the survival of suspension Colo 205 cells (IC_50_ = ~15 ng/mL) by inducing apoptosis, and inhibited the clonogenic activity of Colo 205 CSCs in tumorsphere formation assay and soft agar colony formation assay in the same nanogram per milliliter range. We also discovered that at such low concentrations, BFA effectively induced endoplasmic reticulum (ER) stress response as indicated by the increased mRNA expression of ER stress-related genes, such as glucose-regulated protein 78 (GRP78), X-box binding protein 1 (XBP1), and C/EBP homologous protein (CHOP). Finally, we found that BFA reduced the activity of matrix metallopeptidase 9 (MMP-9). These findings suggest that BFA can effectively suppress the progression of colorectal cancer during the tumorigenesis and metastasis stages. These results may lead to the development of novel therapies for the treatment of colorectal cancer.

## 1. Introduction

Colorectal cancer (CRC) is the third most common cancer; and an estimated 1.2 million new cases and 608,700 deaths per year were reported worldwide in 2008 [1]. It is the second most commonly diagnosed cancer in females and the third in males. CRC is also the fourth most common cause of cancer death [1]. The long-term costs of colorectal cancer, estimated up to be $50,175 per patient in 2009, continue to rise [2]. Colorectal cancer rates have increased rapidly in countries including Taiwan [3], and effective therapeutic solutions are urgently needed [4]. 

Cancer stem cells (CSCs) are a small subset of cancer cells with indefinite potential for self-renewal and the capability to drive tumorigenesis [5]. These cells are believed to be responsible for tumor initiation, progression, metastasis, chemotherapy and radiation resistance, and tumor relapse after therapy [6,7]. Therefore, targeting CSCs is crucial for effective treatment of colorectal cancer [8]. A small percentage of CSCs also persist in established cancer cell lines [9]. In suspension cultures, CSCs resist suspension-induced apoptosis and undergo clonal expansion by forming tumorspheres [10]. A number of potential anti-CSC drugs have been identified based on their ability to inhibit the ability of CSC to survive and form tumorsphere in suspension cultures [11,12].

Brefeldin A (BFA) is a lactone antibiotic first isolated from the fungus *Eupenicillium brefeldianum* [13]. BFA inhibits the transport of proteins from endoplasmic reticulum (ER) to Golgi apparatus and leads to protein accumulating in ER [14]. Prolonged blocking of protein transport by BFA results in ER stress and apoptosis through multiple cellular events including the induction of C/EBP homologous protein (CHOP) [15,16]. Glucose-regulated protein 78 (GRP78) is a molecular chaperone with import functions at the cellular level including the regulation of intracellular calcium, protein folding and ER stress [17]. X-box binding protein 1 (XBP1) is a transcription factor that regulates the functions including cellular stress response. Alternatively spliced XBP1 is a typical indicator of ER stress [18]. The cancer-inhibitory ability of BFA has been preliminarily tested in human cancers such as prostate, leukemia and colon cancer [19]. However, the effects of BFA on cancer stem cells have not been investigated. Here we report, for the first time, the inhibitory effect of BFA on the CSC properties of human colorectal cancer Colo 205 cells.

## 2. Results and Discussion

### 2.1. Colo 205 Suspension Cells Were Sensitive to the Cytotoxic Effect of BFA

**Figure 1:**
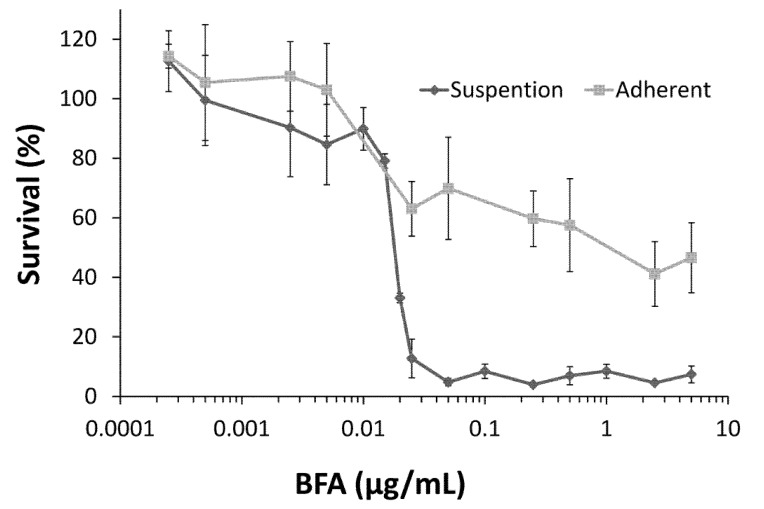
The effect of BFA on the survival of the human colorectal cancer Colo 205 cells cultured under suspension or adhesion conditions. Colo 205 cells (1 × 10^4^/well) grown in regular or ultra-low adhesion 96 well plates were treated with 0 to 5 μg/mL BFA. After 2 days, cell survival was determined by WST-1 assay and normalized to untreated control cells. Data from three independent experiments were presented (mean ± SD, n = 3).

In addition to being capable of undergoing anchorage-independent growth, CSCs from cell lines or tumor samples of colorectal cancer are able to generate tumorspheres in suspension cultures [20,21]. Cytotoxicity toward suspension cultures has been used as a method for the preliminary screening of drugs targeting CSCs [12]. To test the effect of BFA on the human colorectal cancer Colo 205, cells cultured under adhesion or suspension conditions were treated with 0.25 ng/mL to 5 μg/mL BFA for 72 h and their survival was determined by WST-1 reagent. The results in Figure 1 show that, in general, the survival rate of Colo 205 cells decreased with the increasing concentrations of BFA. Importantly, suspension Colo 205 cells were very sensitive to BFA. Most suspended cells died when BFA concentration reached 20 ng/mL, whereas adhesion cells remained about 60% viable until BFA concentration was greater than 5 μg/mL. This data indicates that BFA is cytotoxic for Colo 205 cells grown in suspension cultures with an estimated IC_50_ of ~15 ng/mL.

### 2.2. BFA Reduced the Clonogenicity of Colo 205 CSCs

**Figure 2:**
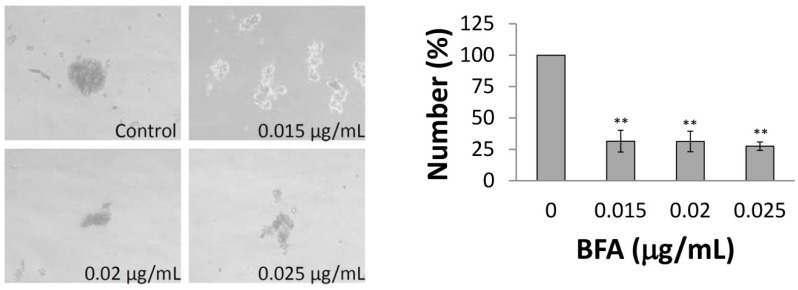
The effect of BFA on the number of Colo 205 tumorspheres. Colo 205 cells (1000 cells /well) were grown in ultra-low attachment 96 wells in the presence of 0.012 to 0.025 µg/mL BFA for two weeks. The images of tumorspheres were captured under phase contrast microscopy. ImageJ was used to determine the number of tumorspheres with diameters larger than 50 µm. Representative results from three independent experiments are presented. (mean ± SD, n = 3; **, *p* < 0.01).

**Figure 3:**
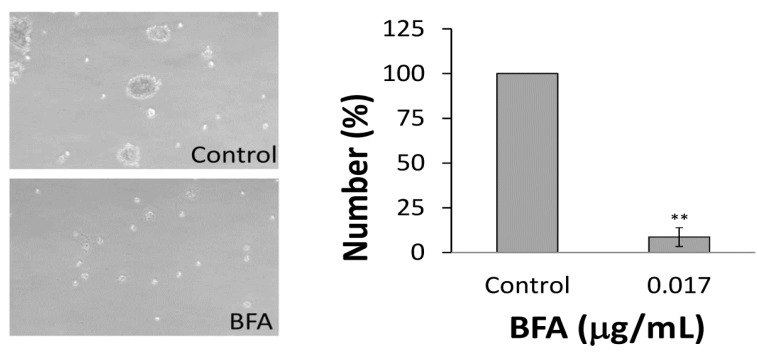
The effect of BFA on Colo 205 colony formation in soft agar. Colo 205 cells embedded in soft agar with or without 0.015 μg/mL BFA in the soft agar were cultured for two weeks. The images of colonies were captured under phase contrast microscopy. ImageJ was used to determine the number of colonies with diameters larger than 50 µm. Representative results from three independent experiments are presented. (mean ± SD, n = 3; **, *p* < 0.01).

Since the CSCs constitute only a small subset of the total cancer cell population, even in the case of cancer cell lines, we further examined whether BFA affected the ability of the Colo 205 CSCs to generate tumorspheres. ImageJ [22] was used to determine the number of tumorspheres with diameters larger than 50 µm. Figure 2 indicates that the number of tumorspheres were significantly reduced to about 30% of control in cells treated with 15–25 ng/mL BFA. Alternatively, the ability of Colo 205 CSCs embedded in soft agar to form 3-dimentional colonies was also tested. Correspondingly, Figure 3 shows that the number of Colo 205 colonies was greatly reduced by 15 ng/mL BFA. These results indicate that the CSC population of the Colo 205 cell line was reduced by BFA at nanogram per milliliter range.

### 2.3. BFA Induced Apoptosis of Suspension Colo 205 cells

**Figure 4:**
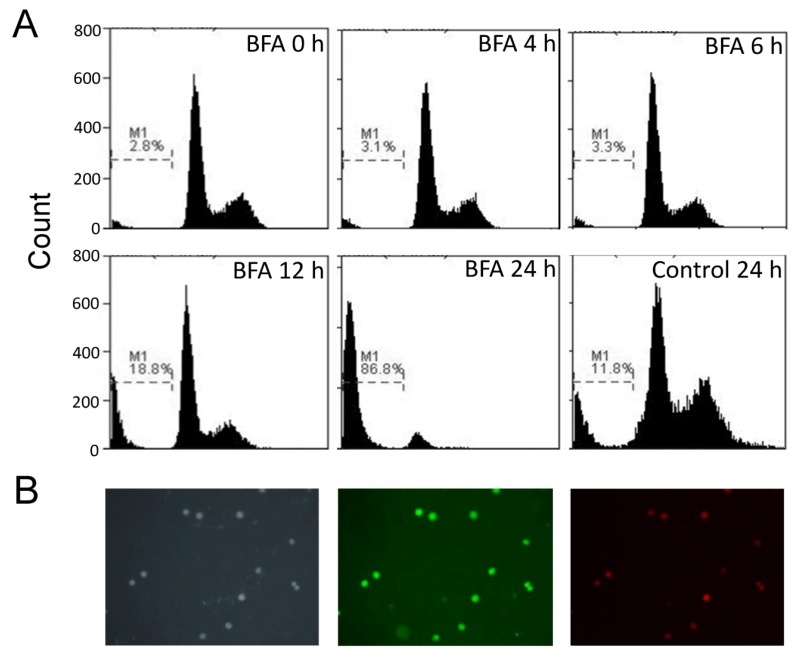
The BFA induced apoptosis of Colo 205 cells in suspension cultures. (**A**) Colo 205 suspension cells were treated with 0.1 μg/mL BFA for 0, 4, 6, 12 and 24 h. The cells were then harvested, fixed and stained with propidium iodide for flow cytometry assay. Sub-G1 region (M1) represented apoptotic cells. (**B**) Suspension Colo 205 cells treated with 0.1 μg/mL BFA for 12 h were stained with DAPI (2-(4-Amidinophenyl)-6-indolecarbamidine dihydrochloride, left panel), Annexin V-FITC (middle panel) and propidium iodide (right panel), and photographed under a fluorescence microscope at 100× magnification. Representative results from two independent experiments were shown.

DNA fragmentation is one of the distinct features of cells undergoing apoptosis. The fractional DNA content in the apoptotic bodies can be estimated at sub-G1 fraction by flow cytometric assay [23]. Therefore, we used propidium iodide staining and flow cytometry to test whether BFA induced apoptosis of Colo 205 suspension cells. The result of Figure 4A show that the ratio of apoptotic cells at 0, 4, 6, 12 and 24 h after treatment of 0.1 µg/mL BFA were 2.8%, 3.1%, 3.3%, 18.8% and 86.8%, respectively, whereas the ratio of spontaneous apoptotic cells among the control cells at 24 h was only 11.8%. Early apoptotic cells can be detected at 12 h as Annexin V^+^/PI^−^ cells (Figure 4B). These results indicate that the cytotoxic effects of BFA on suspension Colo 205 cells are achieved mainly through the induction of apoptosis.

### 2.4. BFA Induced ER Stress Response in Suspension Colo 205 Cells at Nanogram per Milliliter Range

**Figure 5:**
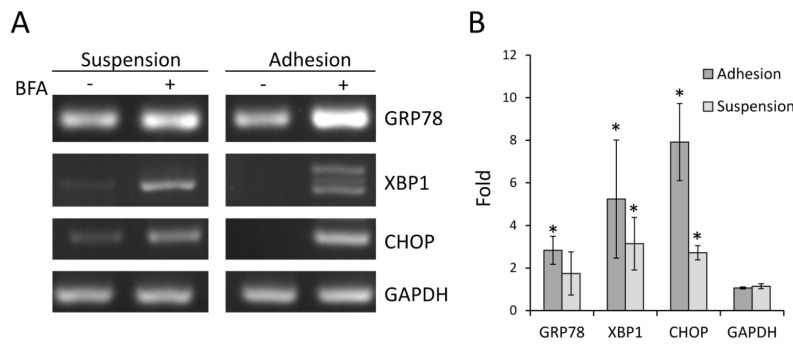
BFA induced the expression of ER stress-related genes in Colo 205 cells. Suspension and adhesion Colo 205 cells were treated with or without 0.015 μg/mL BFA for 24 h. (**A**) The cells were harvested and the total RNA was extracted for RT-PCR followed by 2% agarose gel electrophoresis to detect the expression of GRP78, XBP1 and CHOP. GAPDH were used as the internal control. Representative results from at least two independent experiments were shown. (**B**) Fold induction of GRP78, XBP1 and CHOP was determined by quantitative RT-PCR. (mean ± SD, n = 3; *, *p* < 0.01 compared to GAPDH).

GRP78, XBP1 and CHOP are mediators and markers of ER stress response. Accumulation of unfolded protein inside ER enhances the expression of these genes through transcription regulation mechanisms [24,25], and also induces the alternative splicing of XBP1 [26].To determine whether Colo 205 cells from suspension and adhesion cultures displayed different thresholds to BFA-induced ER stress response, the expression of these genes was detected by RT-PCR. As shown in Figure 5A, in general, the expression of these genes was increased in both suspended and adherent cells treated with 15 ng/mL BFA for 24 h. Intriguingly, although the expression of XBP1 was increased by BFA in both culture conditions, only the adhesion cells underwent XBP1 alternative splicing, exhibiting double bands on the agarose gel-electropherogram. Increased expression of these ER stress markers by BFA was also confirmed by quantitatiove RT-PCR (Figure 5B). These results suggest that suspension Colo 205 cells were not more sensitive to BFA-induced ER stress than adhesion cells; however, the underlying processes of the stress response may be different in cells grown in suspension and adhesion cultures.

### 2.5. BFA Reduced the Activity of MMP9

**Figure 6:**
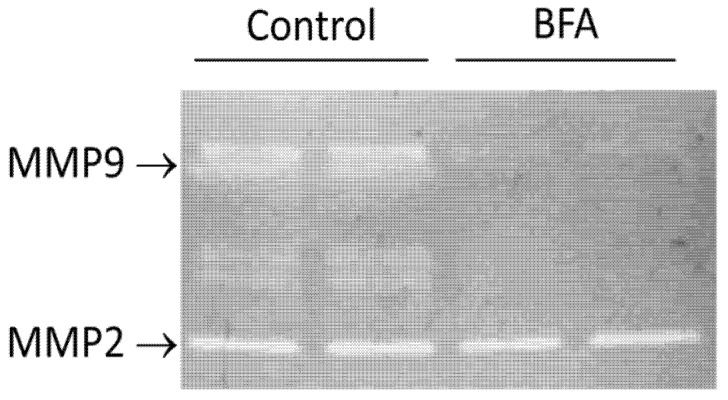
The effect of BFA on the activity of MMPs in Colo 205 cells. Colo 205 adhesion cells were treated with or without 0.015 μg/mL BFA for 24 h. The media were then collected and the MMP activity was analyzed by gelatin zymography assay. Results from duplicate experiments are shown.

Matrix metalloproteinases (MMPs) are proteolytic enzymes involved in various phases of cancer progression, including angiogenesis, invasiveness and metastasis [27]. Knockout of MMP-2 or MMP-9 is found to decrease tumorigenesis [28]. Increased levels of MMPs including MMP-2 and MMP-9 have been reported in the blood and tissue samples from colorectal cancer patients [29]. Recent reports have indicated that CSC characteristics are associated with up-regulated MMP-2 and MMP-9 expression [30]. Therefore, we examined if BFA also reduced the activity of MMPs in Colo 205 cells. Confluent Colo 205 cells were treated with 15 ng/mL BFA in serum-free culture media for 24 h. Then the conditional medium was concentrated by using Centricon filters with 10 kD cutoff and subjected to gelatin zymography analysis. As Figure 6 shows that, while control cells exhibited two major gelatinlytic bands corresponding to MMP-2 and MMP-9, the activity of MMP-9 was greatly decreased in cells treated with BFA leaving MMP-2 unaffected. This result indicates that BFA inhibits the activity of MMP-9 but not MMP-2 of Colo 205 cells. 

### 2.6. Discussion

A growing body of evidence suggests that CSCs play critical roles in the development of cancer, including driving tumorigenesis, promoting metastasis, and evading chemotherapy [31,32]. Here we have investigated the effect of BFA in the CSC properties of human colorectal cancer Colo 205 cells. Our result demonstrated that BFA can inhibit the survival of Colo 205 cells in suspension culture by inducing apoptosis (Figure 1 and Figure 4). BFA inhibited the clonogenesis of Colo 205 both in tumorsphere formation assay and soft agar colony formation assay (Figure 2 and Figure 3). We also demonstrated that BFA induced the expression of ER stress-related genes, such as GRP78, XBP1 and CHOP in both suspension and adhesion cultures of Colo 205 (Figure 5). Finally, we showed that BFA inhibited the activity of MMP-9 (Figure 6). Together these data suggest that BFA is able to inhibit various aspects of CSC characteristics in Colo 205 cells.

Astonishingly, these CSC-inhibitory effects can all be induced by BFA at strikingly low concentrations. The result of Figure 1 showed that the IC_50_ of BFA is about 15 ng/mL, far lower than the microgram per milliliter range usually used for inhibiting ER-Golgi transport in cell biology study. RT-PCR results showed that major markers of ER stress response were indeed up-regulated by 15 ng/mL BFA, therefore we believe that the effects of BFA is likely mediated through the induction of ER stress. However the reason for the differential sensitivity between suspension cells and adhesion cells to BFA remains to be determined. In total, our findings indicate that selective targeting of colorectal cancer stem cells and inhibition of cancer metastasis could be achieved by BFA at low concentration and, therefore, with few side effects. Further studies are warrant to test the potential of BFA as an effective anti-CSC drug for clinical treatment of colorectal cancer.

## 3. Experimental

### 3.1. Cell Culture

Colo 205 cells were cultured in in a 1:1 mixture of DMEM and Ham’s F-12 medium, supplemented with 10% fetal calf serum (FCS) at 37 °C in a humidified atmosphere (5% CO2). To culture suspension cells or to propagate cancer stem cells as mammospheres, Colo 205 cells were suspended in DMEM-F12 supplemented with 0.4% bovine serum albumin, 5 μg/mL bovine insulin, 20 ng/mL basic fibroblast growth factor 2 (bFGF), and 10 ng/mL epidermal growth factor (EGF). For long-term tumorsphere culture, fresh media were added every 3 days.

### 3.2. Cell Survival Assay

The cytotoxicity of BFA (Sigma-Aldrich, Steinheim, Germany) was determined by WST-1 reagent (Roche, Mannheim, Germany) as described before [33]. Briefly, cells in exponential growth will be seeded at 1 × 10^4^ per well in a regular or an ultra-low adhesion 96-well plate for attachment and suspension culture, respectively. After indicated periods of time, the WST-1 solution (10 µL/well) was added and incubated for additional 4 h. The absorbance at 450 nm was measured for each well and the viability of control cells was taken as 100% for normalization.

### 3.3. Soft Agar Colony Formation Assay

Colo 205 cells (5 × 10^4^ per well in a 6-well plate) were mixed 4:1 (v/v) with 2.0% low melting agarose in DMEM growth medium for a final concentration of 0.4% agarose. The cell mixture was placed on top of a solidified layer of 0.5% agarose in growth medium. Cells were fed every 6 to 7 days with growth medium and the number of colonies was counted after two weeks under phase contrast microscopy with low magnification.

### 3.4. Determination of Sub-G1 Population and Annexin V/PI Staining

Briefly, to determine the sub-G1 population by flow cytometry, the cells were trypsinized, washed with PBS and re-suspended in hypotonic lysis solution (100 mM Tris, 154 mM NaCl, 1 mM CaCl_2_ and 0.5 mM MgCl_2_ and 0.1% NP-40) with 50 μg/mL of propidium iodide (PI) and 40 μg/mL of RNase. The cells were incubated in ice for 20 min before being analyzed by flow cytometry. To detect early apoptotic cells, trypsinized and washed cells were stained with Annexin V-FITC and PI using AnnexinV-FITC Apoptosis Detection Kit (Strong Biotech, Taipei, Taiwan).

### 3.5. Reverse-Transcription Polymerase Chain Reaction (RT-PCR)

Total RNA was extracted from cultured cells using Trizol reagent (Invitrogen, Carlsbad, CA) and single step RT-PCR was performed as previously described [34]. Sequences of the oligonucleotide primers are: CHOP: 5'-CAACTGCAGAGAATTCAGCTGA-3' and 5’-ACTGATGCTCTAGATTG TTCAT-3'; GRP78: 5'- GCTCGACTCGAATTCCAAAG-3' and 5'-TTTGTCAGGGGTCTTTCACC-3'; XBP1: 5'-CCTTGTAGTTGAGAACCAGG-3' and 5'-GGGGCTTGGTATATATGTGG-3'; GAPDH: 5'-GAAGGTGAAGGTCGGAGTC-3' and 5'-GAAGATGGTGATGGGATTTC-3'; Actin: 5'-AGAGCTACGAGCTGCCTGAC-3' and 5'-AGCACTGTGTTGGCGTACAG-3'. Fold induction of gene expression was determined by using quantitative RT-PCR and the ∆∆Ct method [35]

### 3.6. Gelatin Zymography

The activities of MMP-2 and MMP-9 were determined as described before [36]. Colo 205 cells were treated with or without 15 ng/mL BFA in serum-free culture medium for 24 h. The medium was then collected and concentrated by using Centricon filters with 10 kD cutoff. Ten µL of 50× concentrated conditional medium were denatured in 2× non-reducing SDS sample buffer and loaded without boiling to a 10% SDS-polyacrylamide gel containing 1 mg/mL gelatin. After electrophoresis, the gels were washed thrice in 50 mM Tris-HCl (pH 7.5) containing 0.15 M NaCl, 5 mM CaC1_2_, 5 μM ZnCl, 0.02% NaN_3_, and 0.25% Triton X-100 at room temperature for 30 min each time, and then incubated in the same buffer without Triton X-100 at 37 °C for 20 h. Finally, the gels were stained by Coomassie Brilliant Blue R-250 solution.

### 3.7. Statistical Analysis

All results are shown as the mean ± SD from three independent experiments. The data were analyzed by Student’s T-test. A *P*-value < 0.05 was considered as statistically significant. Asterisks represent the results of * *p* < 0.05 and ** *p* < 0.01.

## 4. Conclusions

Taken together, results in this study suggest that BFA can effectively suppress the progression of colorectal cancer at tumorigenesis and metastasis steps.
